# Negative symptoms in schizophrenia: clinical framework linking emotional experience and identity

**DOI:** 10.1192/bjb.2026.10261

**Published:** 2026-08

**Authors:** Carol Palma, Susana Ochoa

**Affiliations:** Department of Psychology, FPCEE Blanquerna, Ramon Llull University, Barcelona, Spain; Sant Joan de Déu Research Foundation, Barcelona, Spain

Negative symptoms in schizophrenia are among the most disabling and least responsive dimensions of the disorder. Despite advances in operationalisation, current assessment approaches continue to privilege observable behaviour, often overlooking the subjective emotional experience of patients.^1,2^ This limitation has important clinical implications, as it may lead to incomplete understanding of patients’ difficulties and needs.

A substantial proportion of individuals with schizophrenia experience persistent negative symptoms that interfere with social functioning and autonomy.^3^ Although multidimensional models and second-generation scales have improved assessment,^4^ these tools primarily capture external manifestations such as reduced expressivity or activity. In routine practice, this may obscure the internal processes underlying these symptoms.

From a clinical perspective, negative symptoms involve profound alterations in emotional processing, motivation and goal-directed behaviour. Unlike positive symptoms, which may allow post-episode meaning-making, negative symptoms tend to affect the individual’s ongoing experience of self. Patients frequently report difficulties describing their internal states, including feelings of emotional blunting, mental blockage during social interaction and frustration at being misunderstood. These experiences suggest that negative symptoms are not merely behavioural deficits but are closely linked to disruptions in emotional experience and identity.

To address this gap, we propose a simple clinical framework integrating three interconnected domains: (a) observable negative symptoms (e.g. blunted affect, avolition); (b) subjective emotional experience (e.g. diminished emotional resonance, anticipatory anhedonia); and (c) identity and self-related processes (e.g. reduced agency, disruption in self-continuity). These domains interact dynamically: behavioural withdrawal may reduce emotional feedback, whereas impaired emotional experience may weaken motivation and contribute to identity disruption. This framework aims to support clinicians in moving beyond descriptive assessment towards a more integrative understanding of patient experience.

The distinction between primary and secondary negative symptoms further complicates assessment. In both cases, patients may experience a sense of loss – either of prior functioning or of identity constructs linked to psychotic experiences. In addition, metacognitive deficits may limit patients’ capacity to reflect on and communicate these changes,^5^ reinforcing patterns of withdrawal and reduced engagement. Clinically, this perspective highlights the need to complement standardised tools with personalised interviewing strategies. Exploring emotional suffering and perceived losses, and their impact on self-esteem and identity, may improve diagnostic accuracy and therapeutic alliance. Importantly, reduced verbal expression should not be interpreted as absence of emotional experience without careful exploration.

Incorporating subjective and identity-related dimensions into the assessment of negative symptoms may enhance clinical understanding and inform more targeted interventions. Recognising the emotional burden associated with these symptoms is essential for advancing patient-centred care in schizophrenia.
